# Circulating tRNA-derived fragments in women with endometriosis: a pilot study

**DOI:** 10.1186/s43556-026-00629-8

**Published:** 2026-11-04

**Authors:** Alla Vash-Margita, Hanna Surmann, Ramanaiah Mamillapalli, Hugh S. Taylor

**Affiliations:** 1 Obstetrics, Gynecology and Reproductive Sciences, Yale School of Medicine, 310 Cedar Street, New Haven, CT 06510 USA; 2 Department of Gynecology and Obstetrics, Münster University Hospital, Albert-Schweitzer-Campus 1, 48149 Münster, Germany

Dear Editor,

Endometriosis (EM) is a common gynecological disease affecting ~190 million women worldwide, characterized by the growth of endometrial-like tissue outside the uterus that causes pelvic pain and infertility. Despite its high prevalence, diagnosis is often delayed for years, due to the lack of non-invasive biomarkers. Although various biomolecules have been proposed as diagnostic biomarkers, none are currently used in clinical practice [1]. Therefore, novel biomarkers for early EM diagnosis are urgently needed. Transfer RNA-derived fragments (tRFs) are small non-coding RNAs generated by a specific tRNA cleavage that regulate translation, epigenetic modification, and gene silencing [2]. Although aberrant expression of tRFs has been identified in endometriotic tissues [3], the presence and potential diagnostic value of circulating tRFs have not yet been reported. This is the first pilot study to report tRFs in the blood of women with endometriosis. We identified a distinct circulating tRF signature that differentiates women with endometriosis from those without the disease, highlighting the potential of tRFs as novel noninvasive biomarkers for the diagnosis of endometriosis. Women undergoing laparoscopy for suspected benign gynecologic conditions or chronic pelvic pain were enrolled. Blood was collected on the day of surgery after consent and processed for serum RNA extraction. Controls had no surgical evidence of endometriosis; endometriosis severity was classified using the revised American Society for Reproductive Medicine (rASRM) classification system.

Serum-derived total RNA was used to generate small RNA sequencing libraries. tRF abundance was quantified using a customized analysis pipeline for tRFs, and differential expression was assessed using DESeq2 with FDR correction (adjusted *p* < 0.05). Results were internally validated and compared with edgeR analyses to assess the robustness of identified tRF profiles. We performed an internal validation of differential expression stability and results were validated by comparison with the edgeR quasi-likelihood F-test (RRID:SCR_012802), and concordance of log2 fold-changes was evaluated.

The demographic and clinical characteristics of participants with and without endometriosis are presented in Fig. 1a. A total of 11 participants were included in the study, comprising the endometriosis group (*n* = 4) and the control group (*n* = 7). No significant differences were observed between the endometriosis and control groups in age, BMI, race, hormonal treatment, or menstrual cycle phase (Fig. 1a). Controls had various benign gynecologic conditions other than endometriosis. DESeq2 analysis identified several tRFs that were significantly expressed between the two groups. Following Benjamini–Hochberg correction for multiple testing, 82 tRFs were identified as differentially expressed between women with endometriosis and controls (adjusted *p* < 0.05), including 19 upregulated and 63 downregulated tRFs (Fig. 1b). Significant tRFs were ranked by absolute log2 fold change. Differential expression results were highly concordant between DESeq2 and edgeR (Fig. 1b), with near-identical log2 fold-change estimates (Pearson *r* ≈ 0.99; Spearman *ρ* ≈ 1). While edgeR identified fewer FDR-significant tRFs, the direction and magnitude of effects were consistently preserved across methods. Leave-one-out (LOO) analysis demonstrated that the direction of effect for most significantly altered tRFs remained stable across sample perturbations, indicating that the findings were not driven by individual influential samples. To identify the most significantly altered tRFs, the 10 tRFs with the largest absolute log2 fold-changes (ranging from −3.81 to +4.49) were selected and visualized in a volcano plot (Fig. 1c). These included five upregulated tRFs (tRF-19-WB8US5IU, tRF-21-WR381H93E, tRF-21-WRN81H93E tRF-17-73VL4YK, and tRF-20-9PMNH5IS) and five downregulated tRFs (tRF-27-Q1QU9P9L842, tRF-28-Q1QU9P9L84DF, tRF-27-E1Q89P9L842, tRF-30-PW5SVP9N15WV, and tRF-27-Q1Q89P9L8N2) listed in a table as shown in Fig. 1c. The adjusted *p*-values for these candidates ranged from 0.0014 to 0.0471. Among the 82 significantly differentially expressed tRFs identified in this study, seven were also significantly altered in our previously reported baboon model of endometriosis [4]. Of these, three tRFs were upregulated (tRF-22-WB8US5652, tRF-16-W9RKM80, and tRF-18-3290Q4DZ) and four were downregulated (tRF-27-E1Q89P9L842, tRF-27-Q1Q89P9L842, tRF-27-Q1Q8XP9L842, and tRF-30-E1Q89P9L8422) (Fig. 1d). The concordant expression patterns observed in both human and baboon endometriosis support their potential biological relevance. Receiver operating characteristic (ROC) analyses were performed for the top 10 differentially expressed tRFs identified by DESeq2, with area under the curve (AUC) values calculated from normalized counts using the pROC package in R. ROC analyses demonstrated discriminatory performance, with area under the curve (AUC) values ranging from 0.68 to 0.96. The highest performance was observed for tRF-19-WB8US5IU (AUC = 0.96), followed by tRF-27-E1Q89P9L842 and tRF-21-WRN81H93E (AUC = 0.93) (Fig. 1b). These findings suggest potential diagnostic utility of circulating tRFs for endometriosis, although validation in larger independent cohorts is required.

**Fig. 1:**
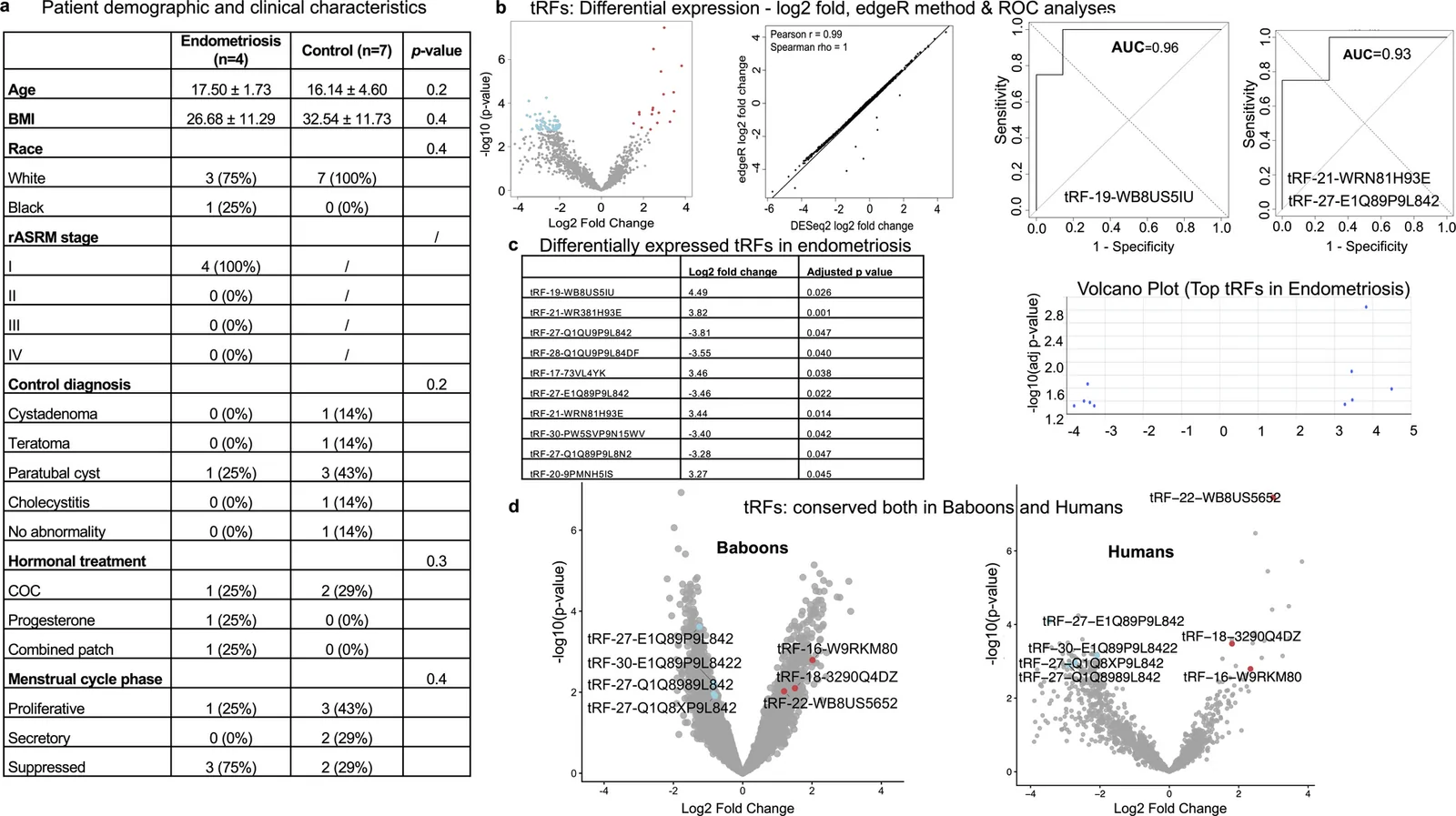
TRFs as potential biomarkers for endometriosis. **a** Demographic and clinical characteristics of participating women. Continuous variables are presented as mean ± standard deviation and compared between the endometriosis and control groups using the Wilcoxon rank-sum test. Categorical variables are presented as number (%) and group differences were assessed with Fisher’s exact test. *p*-values reflect differences between endometriosis and control groups. (RASRM—Revised American Society for Reproductive Medicine classification; COC—combined oral contraceptives). **b** Differential expression and robustness analysis of tRNA-derived fragments (tRFs) using DESeq2 and EdgeR in endometriosis. Volcano plot shows differential expression of tRFs between endometriosis and controls, highlighting distinct tRF expression patterns associated with endometriosis. Each point represents an individual tRF identified using an adjusted *p*-value < 0.05, plotted according to its log_2_ fold change and statistical significance. Upregulated tRFs are shown in red and downregulated tRFs in blue. Robustness analysis comparing effect size estimates generated by DESeq2 and edgeR. The scatter plot displays log_2_ fold changes for individual tRFs calculated using the DESeq2 framework (x-axis) and the edgeR quasi-likelihood framework (y-axis). Each point represents a single tRF, and the solid diagonal line denotes the line of identity (*y* = *x*). Pearson and Spearman correlation coefficients indicate strong concordance between the two analytical approaches in both the direction and magnitude of differential expression, supporting the robustness and reproducibility of the identified endometriosis-associated tRF signatures. Diagnostic performance by receiver operating characteristic (ROC) curve analysis of the top 10 differentially expressed tRFs. ROC curves for individual tRFs were tested, with diagnostic accuracy assessed by the area under the curve (AUC). Among the candidates, tRF-19-WB8US5IU demonstrated the highest diagnostic performance (AUC = 0.96), followed by tRF-21-WRN81H93E and tRF-27-E1Q89P9L842 (AUC = 0.93 for both) suggesting that these three tRFs may serve as promising biomarkers for endometriosis. The remaining seven tRFs exhibited AUC values below 0.90, indicating comparatively lower discriminatory capacity. **c** Table shows the top 10 circulating tRFs differentially expressed in endometriosis compared with controls. TRFs are ranked by absolute log2 fold change among those with Benjamini–Hochberg adjusted *p *< 0.05. Positive log2 fold changes indicate higher abundance in endometriosis; negative values indicate lower abundance. Volcano plot showing the top ten differentially expressed tRFs. Each point represents an individual tRF identified using a Benjamini–Hochberg adjusted *p*-value < 0.05 and ranked by absolute log₂ fold change and is plotted according to its log₂ fold change and statistical significance. The five most significantly downregulated tRFs are highlighted on the left side of the plot, while upregulated tRFs are shown on the right side in blue. The distribution of significantly altered tRFs reveals distinct circulating tRF expression signatures associated with endometriosis. **d** Volcano plots showing conserved circulating tRFs that are differentially expressed across baboons and humans. Differential expression was assessed using DESeq2, with each point representing a conserved tRF plotted according to its log₂ fold change and Benjamini–Hochberg adjusted *p-*value. Significantly altered tRFs shared between species (adjusted *p* < 0.05) are highlighted, with downregulated tRFs shown in blue and upregulated tRFs in red. The consistent direction and significance of these tRF expression changes across humans and baboons demonstrate cross-species conservation of endometriosis-associated circulating tRF signatures

These findings extend previous observations of altered circulating miRNAs in endometriosis [5] and identify tRFs as a previously unrecognized circulating small RNAs in endometriosis. Aberrant tRF expression has been linked to numerous pathological conditions, including multiple cancers, metabolic and neurodegenerative diseases, viral infections, and cellular stress responses [2], suggesting broad regulatory functions in health and disease. In the context of endometriosis, a chronic inflammatory disorder characterized by immune dysfunction, the observed changes in circulating tRFs may reflect systemic molecular responses that contribute to disease pathophysiology and offer novel opportunities for biomarker development. Among the top ten differentially expressed tRFs, only tRF-30-PW5SVP9N15WV has been previously implicated in disease, with elevated levels reported to correlate with poor prognosis in breast cancer. Interestingly, we observed reduced expression of this tRF in endometriosis, indicating that its function may be disease- and tissue-context dependent. The remaining nine top-ranked tRFs have not been previously associated with endometriosis or any other disease, highlighting the largely unexplored nature of the tRF landscape. These findings emphasize the need for mechanistic studies to define the biological functions of tRFs and to determine their utility as biomarkers and potential therapeutic targets in endometriosis. Unlike previous studies focused on endometriotic lesions, this work represents the first comprehensive analysis of circulating serum tRFs in endometriosis, revealing a distinct systemic small RNA signature associated with the disease. The absence of overlap between the specific tRFs identified here and those reported in lesion tissues suggests that local and systemic manifestations of endometriosis may be governed by different tRF populations. Tissue-derived tRFs are likely reflective of processes intrinsic to ectopic lesions, whereas circulating tRFs may represent broader host responses involving inflammatory, immune, and metabolic pathways. Importantly, the accessibility of blood sampling makes circulating tRFs more amenable to the development of noninvasive diagnostic assays than tissue-derived RNA biomarkers, which require surgical specimen collection. The limitations of this study include the small sample size, early-stage endometriosis cases, and the use of controls with other benign gynecologic conditions rather than pathology-free individuals, all of which may have influenced circulating tRF profiles. Another limitation emanates from lack of correction for menstrual cycle stage as this represents an important potential source of variation. In addition, the lack of functional validation precludes conclusions regarding the biological roles of the identified tRFs. Finally, circulating RNA signatures may be affected by systemic physiological and environmental factors. Larger validation studies and mechanistic investigations are therefore warranted.

In conclusion, this pilot study provides the first evidence that circulating tRFs are dysregulated in endometriosis. We identified a distinct serum tRF signature that was robust across analytical approaches and included candidates with promising diagnostic performance. These findings establish circulating tRFs as a previously unrecognized class of biomarkers for endometriosis and warrant validation in larger independent cohorts.

## Supplementary Information

Supplementary Material 1.

## Data Availability

RNA-Seq data was deposited into the Gene Expression Omnibus database at NCBI under accession number GSE343024 and are available at the following URL: https://www.ncbi.nlm.nih.gov/geo/query/acc.cgi?acc=GSE343024
