# Supplementary material for: Circulating tRNA-derived fragments in women with endometriosis: a pilot study
Source: Mol Biomed. 2026 Nov 4;7:238. doi: 10.1186/s43556-026-00629-8 (PMC13737271; doi:10.1186/s43556-026-00629-8)
Supplement: Supplementary file 1 [file 43556_2026_629_MOESM1_ESM.dotx]

**Circulating tRNA-Derived Fragments in Women with Endometriosis: A Pilot Study**

Alla Vash-Margita^1^,†,*, Hanna Surmann^1, 2^, †, Ramanaiah Mamillapalli^1^, †, Hugh S Taylor^1^

^1^Obstetrics, Gynecology and Reproductive Sciences, Yale School of Medicine, 310 Cedar Street, New Haven, CT 06510, United States.

^2^Department of Gynecology and Obstetrics, Münster University Hospital, Albert-Schweitzer-Campus 1, 48149, Münster, Germany.

† Contributed equally

*** Corresponding Authors**

Alla Vash-Margita, MD, ORCID: 0000-0002-3510-3945

Ramanaiah Mamillapalli, PhD. ORCID: 0000.0002.4022.8910

Obstetrics, Gynecology and Reproductive Sciences, Yale School of Medicine

310 Cedar Street, New Haven, CT 06510

Email: alla.vash-margita@yale.edu; ramana.mamillapalli@yale.edu

**Supplementary File S1: Materials and Methods**

***Study design and ethical approval***

This pilot case-control study was conducted at Yale-New Haven Hospital (New Haven, CT, USA) and approved by the Institutional Review Board of Yale University School of Medicine. Written informed consent was obtained from all participants aged 18 years or older. For participants younger than 18 years, written informed assent was obtained together with consent from a legal guardian. Participants were recruited at Yale-New Haven Hospital between March 2018 and December 2021. Informed consent was obtained from all individual participants included in the study or from legal guardians as appropriate, protocol # 1608018237.

***Patient recruitment and eligibility***

Women who were undergoing laparoscopic surgery for suspected benign gynecologic conditions or evaluation of chronic pelvic pain were eligible for inclusion.

*Inclusion criteria:*

- Female
- Undergoing laparoscopic surgery
- Ability to provide informed consent

*Exclusion criteria:*

- Cognitive or physical limitations precluding participation
- Refusal to participate
- Pregnancy

For the present pilot analysis, participants were assigned to the endometriosis group if endometriosis was confirmed by intraoperative findings and/or histopathological evaluation. Control participants had no surgical evidence of endometriosis. The presence of other benign gynecologic findings, including paratubal cysts, was permitted in the control group. Disease severity among participants diagnosed with endometriosis was classified according to the revised American Society for Reproductive Medicine (rASRM) staging system.

***Sample collection and processing***

- Peripheral blood samples were obtained from participants prior to surgical intervention on the day of surgery. A total volume of approximately 5-10 mL of blood was collected into sterile collection tubes (BD, Franklin Lakes, NJ, USA). Following collection, samples were processed promptly to obtain serum. Blood samples were centrifuged at 2,500 rpm for 10 minutes at 4 °C, and the resulting serum fraction was carefully separated. Serum aliquots were stored at -80 °C until RNA extraction and downstream analyses. A total of 11 participants (4 endometriosis and 7 controls) were included in the study from Children’s Hospital at Yale University.

***RNA isolation***

Total RNA was isolated from serum samples using the Qiagen miRNeasy Mini Kit (cat. #17004, Qiagen, Germantown, MD, USA), following the manufacturer’s protocols. The purified RNA was assessed for concentration and purity by measuring absorbance ratios at 260/280 nm and 260/230 nm on a NanoDrop spectrophotometer. RNA integrity was evaluated using either an Agilent Bioanalyzer or Fragment Analyzer to examine ribosomal RNA peak patterns; samples with satisfactory quality metrics (high integrity and appropriate ratios) were selected for downstream library construction.

***RNA library preparation and sequencing***

Small RNA libraries were generated from the extracted RNA using the Qiagen microRNA Library Prep Kit (Cat# 331505). Each sample was normalized to a defined input range prior to library synthesis. Adapters were ligated sequentially to the 3′ and 5′ ends of small RNAs, followed by reverse transcription. Unique molecular identifiers (UMIs) were incorporated during this process to enable accurate read quantification. The resulting cDNA was purified, amplified, and subjected to a final cleanup step to produce sequencing libraries. Libraries were assessed on an Agilent TapeStation or equivalent platform, and if necessary, size selection was performed to remove adapter dimers. Quantification of libraries was achieved using qPCR with a commercially available kit (KAPA Biosystems) to ensure optimal loading concentrations.

Libraries were normalized to 1.2 nM and loaded onto Illumina NovaSeq flow cells in accordance with Illumina’s established protocols. Each lane included a low-level spike-in of a PhiX control library to monitor sequencing performance in real time. Sequencing was performed with 100 bp paired-end reads, and an 8 bp unique dual index was read during dedicated index cycles following the first read. Raw sequencing data were transferred to a high-performance computing environment for downstream analysis. For tRF analysis, read processing and alignment were conducted using an adapted small RNA sequencing pipeline tailored to capture tRF sequences. Reads were trimmed and filtered for quality before mapping to the appropriate small RNA reference. Raw counts representing tRF abundance were generated and used as input for differential expression analysis.

***Statistical analysis***

Small RNA sequencing count data were analyzed using the DESeq2 package (RRID:SCR_000154) in R (version 4.5.0). Prior to differential expression analysis, low abundance tRFs were filtered to reduce noise and improve statistical power. Specifically, tRFs with fewer than 10 reads in at least two samples were excluded from downstream analyses. Differential expression was assessed using a negative binomial generalized linear model as implemented in DESeq2, with experimental group included as the primary factor in the design formula. Size factor normalization was applied to account for differences in library depth across samples. Dispersion parameters were estimated using the local regression fit, which is recommended for datasets with moderate sample size and heterogeneous variance structure. P-values were adjusted for multiple testing using the Benjamini–Hochberg false discovery rate (FDR) procedure. Adjusted *p*-values (padj) < 0.05 were considered statistically significant. We performed an internal validation of differential expression stability using a leave-one-out (LOO) resampling approach, whereby each sample is excluded in turn, and differential expression analysis is repeated to evaluate the consistency of effect directions across runs. To assess robustness, differential expression results were also compared with those obtained using the edgeR quasi-likelihood F-test (RRID:SCR_012802), with concordance of log2 fold changes considered in the interpretation of tRF profiles.

***Comparison to previously published baboon data***

Comparisons with previously identified circulating tRF signatures in a longitudinal baboon model of experimentally induced endometriosis were based on data published before and were not newly generated as part of the present study. Detailed information regarding animal characteristics, induction procedures, sample collection, sequencing methods, and statistical analyses has been reported previously.^1^

1. Surmann, H., Mamillapalli, R., Joshi, N., Burns, G., Fazleabas, A. T., & Taylor, H. S. (2026). Circulating transfer RNA-derived fragment abundance after experimental endometriosis induction in baboons. *Mol Hum Reprod*, *32*(2). <https://doi.org/10.1093/molehr/gaag032>
